# Impaired contraction of blood clots precedes and predicts postoperative venous thromboembolism

**DOI:** 10.1038/s41598-020-75234-y

**Published:** 2020-10-26

**Authors:** Natalia G. Evtugina, Alina D. Peshkova, Arseniy A. Pichugin, John W. Weisel, Rustem I. Litvinov

**Affiliations:** 1 Institute of Fundamental Medicine and Biology, Kazan Federal University, Kazan, Russian Federation; 2 Department of Neurosurgery, Interregional Clinical Diagnostic Center, Kazan, Russian Federation; 3 Department of Cell and Developmental Biology, University of Pennsylvania School of Medicine, 421 Curie Blvd., BRB II/III, Room 1116, Philadelphia, PA 19104 USA

**Keywords:** Vascular diseases, Platelets, Diagnostic markers, Risk factors

## Abstract

Deep vein thrombosis (DVT) is a common but unpredictable complication of surgical interventions. To reveal an association between the blood clot contraction (retraction) and the incidence of postoperative venous thrombosis, 78 patients with brain tumors that were operated on were studied, of which 23 (29%) were diagnosed with postoperative DVT. A clot contraction assay, along with other hemostatic and hematologic tests, was performed 1–3 days before the surgery and on the 1st day and 5–7th days after the surgery. On the 1st postoperative day, clot contraction was significantly suppressed in patients who subsequently developed DVT, compared to the patients without DVT. Importantly, this difference was observed at least 5 days before DVT had developed. The weakening of contraction on the 1st postoperative day was more pronounced in the DVT patients with malignant versus benign brain tumors, atherosclerosis, hypertension, as well as in patients receiving steroids before and during the operation. These results indicate that impaired clot contraction in the postoperative period is associated with imminent DVT, suggesting that it is a prothrombotic risk factor and promotional mechanism. The clot contraction assay has a predictive value in assessing the threat of postoperative thrombosis in patients with benign and malignant brain tumors.

## Introduction

Venous thromboembolism (VTE) that includes deep venous thrombosis (DVT) and pulmonary embolism (PE) is a major complication after surgery and represents a large social and medical problem^1^. Without preventive measures, DVT develops in 10–40% of general surgical patients^2^. The risk of postoperative VTE is especially high in patients undergoing neurosurgical procedures, and has been reported to vary from 6 to 43%^3^ or 19–50% for DVT and up to 25% for PE^4^. VTE develops considerably more often in neurosurgical patients with neoplastic diseases^5–9^, such as meningioma^10^ and high-grade glioma^11^. In patients with brain tumors, DVT was reported to occur in about 30% of cases; while only 1.5–5% of patients with DVT develop PE, the fatality rate in PE is up to 50%^2,3,12^. In addition to common risk factors for surgery, such as venous stasis from postoperative immobility, tissue injury, and inflammation, malignancy is an additional potent risk factor for VTE development^13^. In general, VTE in patients undergoing craniotomy for primary malignant brain tumors remains a major cause of morbidity and mortality^11,14–16^.

Given the risk of postoperative VTE, most patients undergoing general surgery receive prophylaxis that includes unfractionated heparin or low-molecular weight heparin, which reduce the incidence of DVT and PE^17^. However, in neurosurgical patients, anticoagulation increases the risk of intracranial postoperative hemorrhage, one of the most frequent and severe complications^18^, especially in patients undergoing operations for brain tumors^7–9,19^. Intracranial hemorrhage is the most common reason for reoperation within the first days after brain surgery^20^. Therefore, early administration of heparin after craniotomy is unsafe and quite seldom used for prophylaxis of postoperative VTE. Notably, as an alternative to pharmacological DVT prophylaxis with heparin, aspirin, or oral anticoagulants, mechanical methods can be preferably applied, such as exercises, elastic stockings, and intermittent pneumatic calf compression, as well as electrical stimulation^21^.

An important measure to manage thrombotic complications and improve outcomes in patients undergoing neurosurgical intervention is a preoperative analysis of the risk of postoperative DVT. Several clinical factors have been shown to be somewhat predictive of the postoperative VTE risk and have helped to identify postcraniotomy patients who particularly need postoperative precautionary measures^22^. These patients include those with older age, higher BMI, malignant tumor or hypertension^23^. Also, there is a direct correlation between the operation time and DVT incidence^12^. Preoperative steroid usage, male gender, and comorbidities can be also associated with a high risk of DVT and PE^20^. In addition to these clinical factors that cannot be used as a reliable and specific predictor for VTE in (neuro)surgical patients, we asked if there were laboratory blood tests that could have a relatively strong and justified predictive value for postoperative DVT, based on the pathogenic mechanisms of thrombosis.

Virchow’s triad characterizes the most general causes of VTE as a combination of blood hypercoagulability, changes of blood rheology, and local damage to the endothelium^24^. Hypercoagulability, resulting from pathological changes in blood composition before and during the operation, is systemic activation of the blood clotting factors that can be revealed as shortening of the plasma clotting time in vitro*,* often associated with a high fibrinogen level. However, these tests, although indicative of a prothrombotic state, are not specific and sensitive enough to discriminate between the low and high risk of thrombosis. Detection of D-dimer in blood is commonly used to rule out or confirm formation of a thrombus, but this fibrin fragment is produced when a blood clot has already formed and dissolved in the body; therefore, the D-dimer test is unlikely to have a predictive value for potential DVT.

The role of platelets is perhaps one of the least explored aspects of VTE; however, there is evidence that platelets participate actively in the formation, as well as in maturation or remodeling of a thrombus^25,26^. In addition to procoagulant properties, platelets are involved in the volumetric shrinkage of blood clots formed either in vitro on in vivo, which is known as clot contraction or retraction^27^. This process is driven by platelet contractile proteins that generate a mechanical traction force, pulling on fibrin to compact the clot^28^. Recently, a reduced extent and rate of clot contraction has been identified in the blood of VTE patients as well as in patients with ischemic stroke and other (pro)thrombotic conditions when compared with clots formed from the blood of healthy subjects. The reduced ability of clots to contract correlates with platelet dysfunction associated with thrombosis, because in VTE, the patient’s platelets are continuously activated as a result of thrombinemia and become exhausted and refractory to an activating stimulus^29^. These findings suggest that pathological changes in the molecular and cellular blood composition that predispose to thrombosis are critical determinants of the lesser ability of clots to contract. Therefore, we hypothesized that an assay for clot contraction in vitro may have a positive prognostic value for obstructive thrombosis, including postoperative DVT. We anticipated that the reduced clot contraction before or immediately after the operation would directly correlate with the incidence of DVT in the early postoperative period, while normal contraction would be associated with an uncomplicated postoperative course.

To test this assumption in clinical settings, we have chosen a cohort of neurosurgical patients with neoplasms for the following reasons. (i) Neurosurgical operations followed by immobility, especially in patients with brain tumors, are strong provocative factors for DVT, with a relatively high incidence of postoperative DVT. (ii) Despite a high risk of thrombosis, patients undergoing craniotomy are the only known cohort of surgical patients that normally do not receive prophylactic anticoagulation in the first few days of the postoperative period to avoid intracranial hemorrhage, which makes the results of hemostasis tests, including the clot contraction assay, pharmacologically unaffected. The specific aim of this study was to follow the dynamics of clot contraction and other hemostatic parameters before and after the operation and compare them in two main clinical subgroups: the neurosurgical patients without and with DVT developed during the first postoperative week.

## Results

### Clot contraction kinetics in the blood of patients without or with postoperative DVT

**Figure 1:**
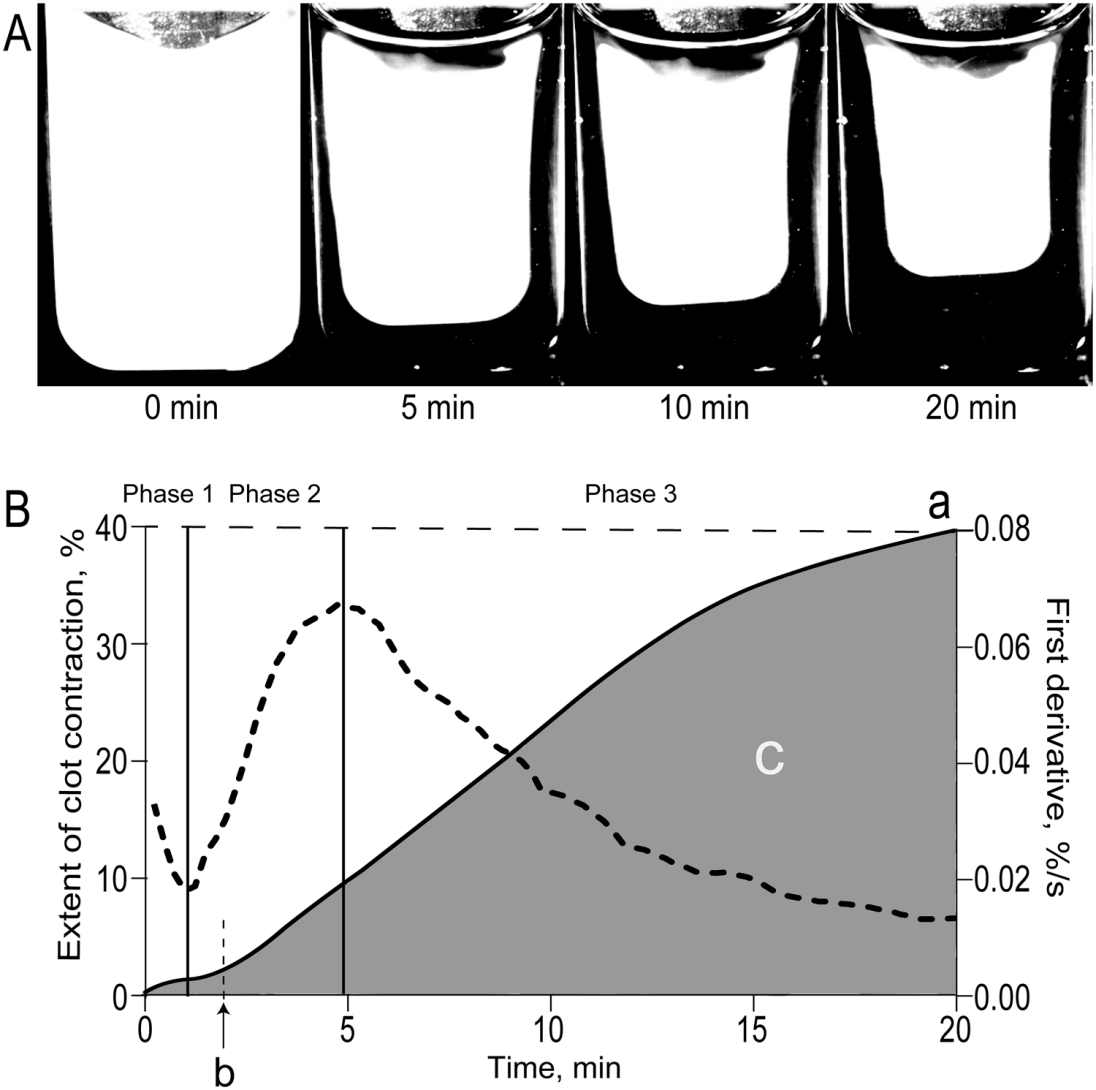
The optical tracking system used for measurements of clot contraction. (**A**) The change in clot size during the process of clot contraction is registered automatically every 15 s by the CCD camera used to measure light scattering. (**B**) The changes in relative clot size are converted computationally into a kinetic contraction curve that can be analyzed for (**a**) the extent of clot contraction at 20 min, (**b**) the lag time or the time to 5% contraction, and (**c**) the area under the curve, which characterizes the entire process of clot contraction and integrates the extent and rate of contraction and the lag time. Using the local maxima and minima of the first derivative (dashed line), the kinetic curve is segregated into three phases, corresponding to initiation of contraction (phase 1), linear contraction (phase 2), and mechanical stabilization (phase 3).

Because DVT develops a few days after surgery, we sought to determine if changes of the blood clot contraction parameters would occur immediately after the surgery and therefore *precede* the thrombotic complications. The clot contraction assay (Fig. 1) was performed in each patient at three time points, namely 1–3 days before surgery, on the 1st day and 5–7th days after the surgery. The results at each time point were compared between two subgroups of patients, those with and without retrospectively diagnosed postoperative DVT.

**Figure 2:**
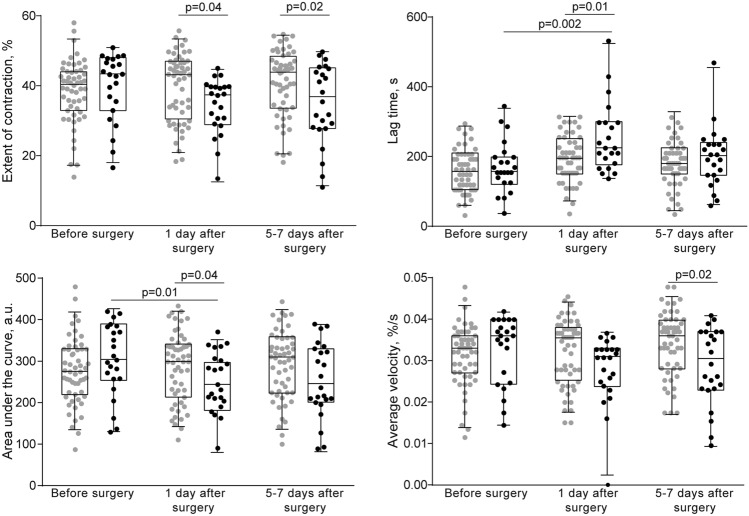
Parameters of blood clot contraction in neurosurgical patients before and after surgery. Gray dot/box plots—patients without postoperative DVT (n = 55); black dot/box plots—patients with postoperative DVT (n = 23). The results are presented as the median and intervals between the 25th and 75th, as well as between the 5th and 95th percentiles (2-way ANOVA with Tukey's multiple comparisons post hoc test).

Unlike pre-surgical values, the postoperative dynamics of the clot contraction parameters diverged depending on the incidence of DVT (Fig. 2 and Supplementary Fig. S1, Supplementary Tables S1 and S2). On the 1st postoperative day, clot contraction was significantly suppressed in patients with subsequent postoperative thrombosis, compared to the patients without DVT, as judged from statistical hypothesis testing (Fig. 1, Supplementary Table S1) or 95% CIs (Supplementary Fig. S1, Supplementary Table S2). Importantly, the difference in clot contraction parameters between patients without and with postoperative DVT was observed on the 1st day after surgery *in the absence* of thrombosis, a few days before clinical signs of DVT emerged. The significantly reduced contraction in the subgroup of patients with DVT remained 5–7 days after the surgery, at or after the time of DVT development (Fig. 2 and Supplementary Fig. S1, Supplementary Tables S1 and S2), which confirmed the known association of the clot contraction assay with ongoing venous thrombosis^29^.

**Figure 3:**
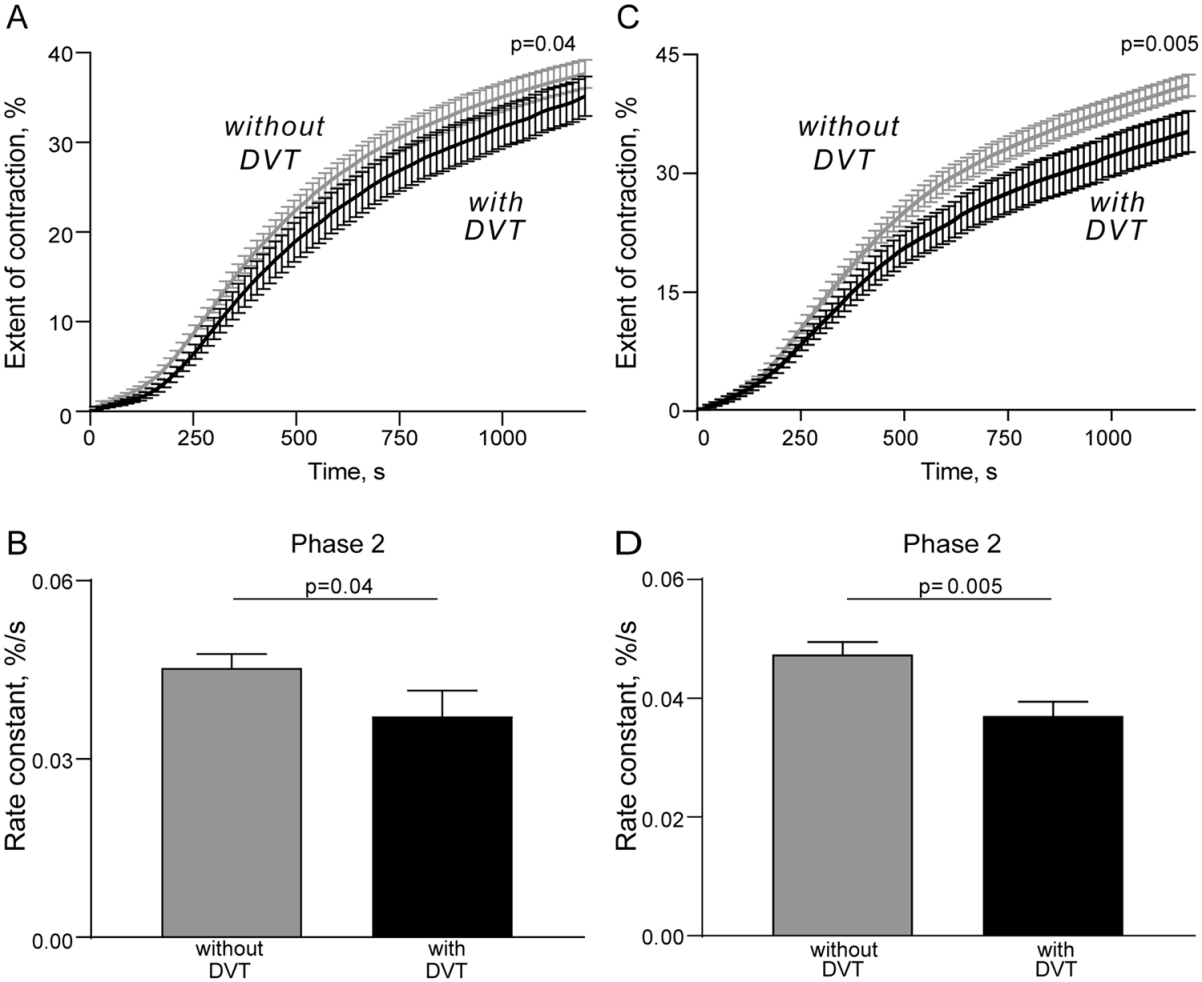
Comparative phase analysis of the averaged kinetic curves of clot contraction for patients without and with postoperative DVT examined 1 day (left) and 5–7 days after the operation. On the averaged kinetic curves (**A,C**), transitions between phases of contraction were determined by finding local maxima and minima within the instantaneous first derivatives (see Fig. 2B). Curves were fit using a piecewise function, and the rate constant of each phase were determined^26^. Average rate constants of Phase 2 (**B,D**) are shown for patients without and with postoperative DVT (Mann Whitney U test).

It has been previously shown that clot contraction occurs in three phases: initiation of contraction (phase 1), linear contraction (phase 2), and mechanical stabilization (phase 3)^26^ Regression analysis conducted on the averaged kinetic curves (Figs. 1B, 3A,C) revealed that in patients with postoperative DVT analyzed at the 1st and 5–7th days after the surgery, the rate constant of phase 2 was significantly reduced compared to patients without DVT (Fig. 3B,D), indicating impairment of the mechanisms of compaction of the clots. There were no significant differences in the rates of phases 1 and 3 between patients without or with postoperative DVT, suggesting that there are no defects in the initiation and stabilization of contracting clots in DVT patients. Again, the variances in the phase kinetics between the patient subgroups appeared only in the postsurgical period, while before the operation there were no differences (Supplementary Fig. S2).

Taken together, the results clearly show that impaired clot contraction in the early postsurgical period is associated with imminent DVT.

### Relation of clot contraction to clinical characteristics of neurosurgical patients

Some clinical features show association with the degree of suppression of blood clot contraction. On the 1st day after surgery, a significant impairment of clot contraction was observed in prospective DVT patients with malignant, in contrast to benign, brain tumors, occlusion and stenosis of precerebral arteries, hypertension, as well as in patients receiving steroids before and during the operation (Supplementary Table S3). The craniotomy by itself was a strong provocative factor for impaired clot contraction and DVT, but without a detectable difference with respect to the duration of the operation. On days 5–7 post operation, occlusion and stenosis of precerebral arteries and steroids were still contributing to the weakening of clot contractility associated with postoperative DVT (Supplementary Table S4). The higher incidence of postoperative DVT could not be attributed to pre-operative inflammation or associated with alterations of the parameters of clot contraction at baseline. In patients with heart failure and COPD (Table 1) there were no pre-existing changes of clot contraction.

**Table 1:** Clinical characteristics of the neurosurgical patients enrolled in the study and segregated into 2 subgroups: without and with postoperative venous thrombosis.

Clinical characteristics	Total number of operated patients, n = 78	Number of patients in clinical subgroups	
		Without postoperative DVT, n = 55	With postoperative DVT, n = 23
Age, years	59 ± 1	57 ± 1	63 ± 2
**Gender**			
Women	59 (79%)	39 (71%)	20 (87%)
Men	19 (21%)	16 (29%)	3 (13%)
**Primary diagnosis**			
Brain tumors			
*Malignant tumors:*	22 (28%)	16 (29%)	6 (26%)
Astrocytic and oligodendroglial	11	8	3
Lymphoma	2	2	–
Ependymal	1	1	–
Mesenchymal	1	1	–
Metastatic	7	4	3
*Benign tumors:*	56 (72%)	39 (71%)	17 (74%)
Meningioma	40	24	16
Neurinoma	12	12	–
Astrocytic and Oligodendroglial	3	2	1
Adenoma	1	1	–
**Comorbidities**			
Occlusion and stenosis of precerebral arteries	61 (78%)	43 (78%)	18 (78%)
Hypertension	32 (41%)	22 (40%)	10 (43%)
Heart failure	21 (27%)	11 (20%)	10 (43%)*
Chronic obstructive pulmonary disease	6 (8%)	2 (4%)	4 (17%)*
Diabetes	9 (12%)	7 (13%)	2 (9%)
**Risk factors for thrombosis**			
History of cerebral ischemia	6 (8%)	3 (5%)	3 (13%)
Chronic ischemic heart disease	3 (4%)	1 (2%)	2 (9%)
**Operative time**			
< 4 h	55 (71%)	39 (71%)	16 (70%)
> 4 h	23 (29%)	16 (29%)	7 (30%)
Immobilization for more than 4 days	5 (6%)	2 (4%)	3 (13%)
Obesity (BMI > 30 kg/m^2^)	20 (26%)	13 (24%)	7 (30%)
Steroids before and during operation (dexamethasone, hydrocortisone)	36 (46%)	24 (44%)	12 (52%)

*p < 0.05 between the patients without and with postoperative DVT (χ^2^-test).

### Cut-off level, sensitivity, specificity, and predictive values of the clot contraction assay for postoperative DVT

**Figure 4:**
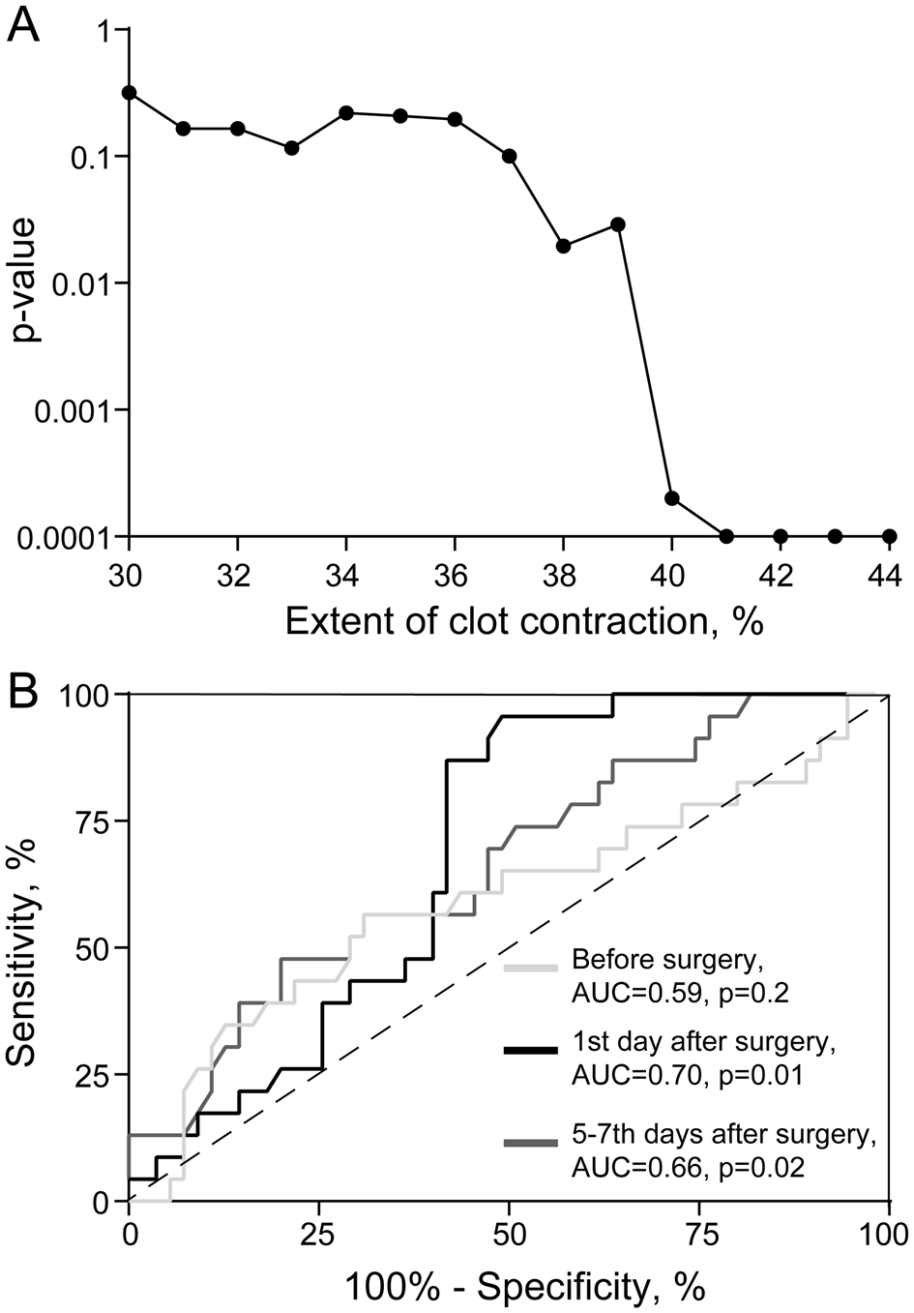
Determination of the cut-off level for the extent of contraction as well as accuracy and significance of the clot contraction assay for predicting postoperative DVT. **(A)** Optimal cut-off level determined by a chi-square test (*p* value on the Y-axis) for the differences in the number of patients without and with postoperative DVT with the extent of clot contraction below or above various cut-off levels (shown on the X-axis). (**B**) ROC curves and AUC values for the extent of clot contraction before surgery, 1st day and 5–7th days after surgery.

On the 1st postoperative day, the extent of clot contraction varied from 18 to 56% in patients without future postoperative DVT and from 11 to 43% in patients with DVT. To define objectively the cut-off level (the borderline between normal and reduced clot contraction) within the overlapping ranges, we first performed a statistical analysis with the chi-square test, in which patients without and with postoperative DVT were segregated into categories that had the extents of clot contraction below or above various cut-off levels defined arbitrarily between 30 and 44%. The normal borderline would be determined as the cut-off level, at which the patient segregation becomes highly significant. Figure 4A shows that the statistical significance, which is related inversely to the *p* value, went up abruptly at a 40% extent of contraction (*p* = 0.0002) and stabilized at the level of 41% and higher (*p* = 0.0001). Therefore, the cut-off level of the extent of clot contraction is 41%.

An independent approach to define the normal borderline is calculating sensitivity, specificity, and predictive values of the clot contraction assay for postoperative DVT at various cut-off levels. From the numbers in the Supplementary Table S5, it can be inferred that the optimal parameters (the highest sensitivity, positive predictive value, and positive likelihood ratio, combined with high specificity and negative predictive value, as well as low positive likelihood ratio) are at the 41–42% cut-off levels of the extent of contraction for a disease prevalence of 30%.

With this borderline, before the operation, the fractions of patients with *reduced extent of contraction* (< 41%) were similar in the subgroups without (49%) and with (35%) postoperative DVT (χ^2^-test, *p* = 0.247), while on the 1st postoperative day, the difference was very highly significant (*p* = 0.000984), so that for 19 of 23 (92%) patients with future DVT, the extent of contraction was below normal, while the number of patients with reduced contraction remained close to baseline in those who did not develop DVT (42%).

Alternatively, these results could be confirmed from the opposite side by revealing the number of patients with *normal extent of contraction* (> 41%). Thus, normal contraction on the 1st day after surgery was observed in 34 of 55 (62%) patients who did not develop future DVT and only in 3 of 23 (13%) who did develop future DVT (χ^2^-test, *p* < 0.00001). This difference remained significant on the 5–7th day after surgery with normal contraction for 41 of 55 (75%) patients who did not develop DVT and 10 of 23 (43%) who did develop DVT (*p* = 0.00843).

To characterize the accuracy of the clot contraction assay, the area under the curve (AUC) of ROC curves was analyzed (Fig. 4B) for the extent of clot contraction. AUC in the clot contraction assay before surgery and on postoperative days 1 and 5–7 were 0.59 (*p* = 0.2), 0.70 (*p* = 0.01), and 0.66 (*p* = 0.02), respectively. These results indicated that the extent of clot contraction at the 1st postoperative had the highest accuracy and significance for predicting postoperative VTE in neurosurgical patients.

### Other hemostatic and hematologic parameters in patients without or with postoperative DVT

To see if other laboratory hemostatic tests could predict and/or reflect development of postoperative DVT, we analyzed the pre- and postoperative dynamics of routine hemostatic parameters presented in the Supplementary Table S6. There was no significant difference in most of the values before and after surgery. Most importantly, even after the surgery, neither aPTT, nor fibrinogen level, nor thrombin time, nor prothrombin time, nor D-dimer showed any distinctions in patients without or with DVT. The average D-dimer level was significantly higher 5–7 days after operation in the DVT patients (Supplementary Table S5), confirming the known association of elevated D-dimer with venous thrombosis developed. Altogether, the results demonstrate that on the 1st postoperative day, none of the hemostatic tests studied, including the D-dimer assay, have a prognostic value for the incidence or absence of postoperative DVT.

Because contraction of blood clots depends strongly on blood composition, we performed hematologic tests in the same patients and at the same time points as for the clot contraction assay (Supplementary Table S7). None of the hematological parameters revealed any significant variations, irrespective of the occurrence of postoperative DVT. It is noteworthy that neutrophil counts inversely correlated with clot contraction parameters, namely with the extent of contraction (*r* = − 0.426, *p* < 0.01), area under the curve (*r* = − 0.417, *p* < 0.05), and average velocity (*r* = − 0.44, *p* < 0.01), suggesting a suppressive effect of the inflammatory cells on clot contraction.

## Discussion

Impaired contraction of in vitro blood clots was found in earlier studies of patients with venous thromboembolism^29^. However, the causality remained unclear as to whether the reduced ability of blood clots to contract is a prothrombotic factor or a consequence of thrombosis. The difficulty in dissecting the cause and effect is that unprovoked thrombosis is unpredictable, while provoked thrombosis is usually prevented pharmacologically, which would distort parameters of the clot contraction assay. Here, clot contraction was studied in neurosurgical patients with brain tumors undergoing craniotomy, who are prone to postoperative venous thrombosis but are not prophylactically anticoagulated due to a high risk of intracranial hemorrhage. Importantly, the lack of heparin administration precluded pharmacological perturbation of the results of the clot contraction assay and other hemostatic tests. The clot contraction assays were performed in the same patients before and the next day after surgery, before the possible occurrence of postoperative DVT. The incidence of the postoperative venous thrombosis in our population was 29%, corresponding to the published rates^2–4,12^.

The study was designed considering two possible outcomes. One is that parameters of clot contraction determined on the 1st postoperative day would be the same in all patients irrespective of whether they later develop DVT or not, while they would be expected to be altered on the 5–7th days after surgery in the patients with DVT. This scenario would mean that changes in clot contraction are concomitant or secondary to thrombosis. The alternative scenario was that the parameters of clot contraction on the 1st postoperative day would be significantly different in the patients with impending DVT compared to the patients without future thrombotic complications. In this case, the impaired clot contraction would precede postoperative thrombosis and, therefore, the clot contraction assay might have a prognostic value for the imminent DVT. Moreover, in the latter case, impaired contraction of in vitro blood clots could be considered a prothrombotic risk factor. This second situation is exactly what has been observed in our study.

The main finding is that all four parameters of the clot contraction assay are significantly altered on the 1st day after surgery in the DVT patients compared to patients without future postoperative DVT (Fig. 2 and Supplementary Fig. S1, Supplementary Tables S1 and S2). We conclude that the impaired blood clot contraction is a sign of forthcoming thrombosis, which suggests using the clot contraction assay as a prognostic tool for postoperative venous thromboembolism.

To assess the performance of the clot contraction assay for predicting postoperative DVT, the lower normal border for the extent of clot contraction was determined to be at the level of 41–42% (Fig. 4A, Supplementary Table S5), which is restricted by the volume fraction of incompressible red blood cells (hematocrit). The overall predictive power of the clot contraction assay of the 1st postoperative day is significant (Supplementary Table S5). The positive predictive value means that among those who had a “positive” clot contraction assay (i.e., the extent of contraction is ≤ 41%) the probability of postoperative DVT is 48%, suggesting that additional examination is required to obtain a more accurate assessment of the risk of postoperative DVT, and prophylactic measures should be applied (perhaps other than anticoagulation, e.g. intermittent pneumatic calf compression). It is noteworthy that this value is limited by the relatively low prevalence of postoperative DVT (~ 30%). The prognostic strength of the clot contraction assay is even more in its negative predictive value, which means that if the result for an individual is negative (i.e., the extent of contraction is > 41%), then the probability of a postsurgical thrombotic complication is ruled out with 95% confidence, so no further testing is necessary.

The positive (LR +) and negative (LR-) likelihood ratios show the ratio between the probability of a positive or negative test result, given the *presence* of the disease and the probability of a positive or negative test result given the *absence* of the disease. The LR + 2.2 means that in the case of a positive result, the post-test probability of DVT is 2.2-fold higher than the pre-test probability, which confirms the utility of the clot contraction assay to assess the risk of postoperative thrombosis. Accordingly, the LR- 0.1 indicates the usefulness of a negative result of the clot contraction assay to rule out the likelihood of postoperative DVT (Supplementary Table S5).

The ROC curve analysis (Fig. 4B) is another way to evaluate the performance of a prognostic or diagnostic test^30,31^ by measuring an AUC value that varies between 0.5 (no discrimination between the absence of presence of a pathological condition) and 1.0 (a perfectly accurate test). The value of 0.7 obtained is considered fair or acceptable and therefore confirms that the extent of clot contraction on the 1st postoperative day has considerable discriminating ability to predict the occurrence or absence of postoperative DVT.

D-dimer was significantly elevated only on the 5–7th postoperative day in the patients with postoperative DVT (Supplementary Table S6), as would be expected from the nature of D-dimer, originating from fibrinolytic cleavage of crosslinked fibrin^32,33^.

The reduced clot contraction in the postoperative period has at least two major underlying mechanisms that at the same time may be associated with a high prothrombotic potential and promote postoperative DVT. The first one is platelet dysfunction resulting from hypercoagulability, leading to continuous activation of circulating platelets followed by their exhaustion and partial loss of ATP-dependent contractility. This mechanism for impaired clot contraction has been demonstrated not only in arterial and venous thrombosis^29,34^, but also in prothrombotic conditions without actual thrombotic events^35^. Our results suggest that the markedly reduced platelet contractility on the 1st postoperative day reflects the pronounced and persistent thrombinemia, resulting from surgical brain tissue damage as a source of tissue factor combined with the relative insufficiency of the physiological anticoagulant activity. It is also likely that the surgery patients with subsequent DVT had enhanced thrombin generation and were initially predisposed to thrombosis due to acquired or hereditary thrombophilia, such as prothrombotic polymorphisms. Irrespective of the underlying mechanisms, the abnormally high postoperative thrombin activity known to cause platelet dysfunction^36^ is a strong prothrombotic factor, which, in combination with two other components of Virchow’s triad (impaired blood flow and endothelial damage), can promote the postoperative DVT.

The second proposed general cause of the impaired clot contraction and subsequent venous thrombosis is pathologic alteration of the blood composition (other than abnormal blood cell counts and fibrinogen level), that causes abnormal clotting likely resulting from enhanced thrombin generation, which modulates the structure and properties of clots and thrombi. In particular, postoperative hypercoagulability and thrombin generation might result in formation of a more dense in vitro blood clot with thinner fibers and a higher mechanical stiffness when the clot contraction assay is performed. Such clots, if formed in vivo, can be also more resistant to fibrinolysis and therefore promote thrombosis through lytic stability^37^. The effect of elevated neutrophil count and activity on clot contraction has never been studied, but cannot be excluded, since the reduced platelet contractility as a part of inflammatory reactions has been demonstrated in inflammatory immune disorders, such as systemic lupus erythematosus^38^ and asthma^39^. This presumption is strongly supported by a significant inverse correlation of the clot contraction parameters and neutrophil counts revealed in our study. Increased hematocrit and fibrinogen levels known to suppress clot contraction^26^ and promote thrombosis could not account for the impaired clot contraction on the 1st postoperative day, because their variations were insignificant (Supplementary Table S6). Certain clinical conditions, such as malignancy, atherosclerosis, hypertension, operations, and administration of steroids, can aggravate the defects in clot contraction and comprise important risk factors for postoperative DVT in neurosurgical patients.

Irrespective of the underlying mechanisms, the impaired contractility of blood clots may *in itself* have prothrombotic potential and promote postoperative DVT in a number of ways. First, the reduced clot contraction may lead to a reduced susceptibility of newly formed intravascular clots and thrombi to internal (patho)physiological fibrinolysis^40^. Second, uncontracted or poorly contracted thrombi must be more obstructive and greatly impair the blood flow in the occluded vessel^34^. Altogether, the defective contractility of blood clots may contribute to increased stability and obstructiveness of an incipient thrombus.

## Conclusions

In the blood of neurosurgical patients with brain tumors, clot contraction was significantly impaired on the 1st postoperative day in those patients who developed postoperative DVT, compared to the patients without DVT. Importantly, the defective clot contraction was observed in the absence of thrombosis. The reduced contraction remained 5–7 days after the operation, at the time when DVT was diagnosed. The impairment of clot contraction on the 1st day after the surgery was most strongly associated with malignant brain tumors, atherosclerosis, hypertension, as well as with administration of steroids before and during the operation. The lower normal limit of the extent of contraction was established at 41%, below which the clot contraction assay performed at the 1st postoperative day had a prognostic value for imminent postoperative DVT. The reduced parameters of clot contraction on the 1st postoperative day correlated inversely with neutrophil counts, suggesting a suppressive effect of postsurgical inflammation on clot contraction. Taken together, these results indicate that impaired blood clot contraction in the early postsurgical period is associated with imminent DVT. This association suggests that reduced contractility may be a prothrombotic risk factor and a pathogenic mechanism. From a practical standpoint, the results of the clot contraction assay can complement other clinical, laboratory, and instrumental data in assessing the risk of postoperative venous thrombosis and suggest the necessity of medicinal and/or non-medicinal thromboprophylaxis.

## Material and methods

### Clinical material and ethical aspects

78 consecutive patients with brain tumors who underwent surgery in the Department of Neurosurgery at the Interregional Clinical Diagnostic Center, Kazan, Russia, during 2017–2019 were enrolled in the study. The study was approved by the Ethical Committee (Reference #70 as of 30.01.2016) of the Interregional Clinical Diagnostic Center, and written informed consent was obtained from the patients. All examinations were performed in accordance with the approved guidelines. The study was performed following the Declaration of Helsinki. All data from patients were treated anonymously. The investigation data were saved in an electronic database with restricted access.

All patients were clinically assessed by a vascular surgeon before the operation to exclude those with a predisposition to DVT, and hemostasis tests were performed to exclude coagulopathy. The exclusion criteria were as follows: a previous episode of objectively documented DVT or PE; signs or symptoms suggestive of current DVT or PE; a history of stroke; an age less than 18 years. Patients were excluded from this study if for any reason they took anticoagulants, thrombolytics or antiplatelet drugs at least 14 days prior to examination. Clinical characteristics of the patients are presented in Table 1. Postoperatively, all the patients received non-pharmaceutical DVT preventive treatments, like elastic stockings and intermittent pneumatic calf compression. No anticoagulants were administered after the surgery until DVT was diagnosed. The postoperative immobility lasted from 2 to 10 days.

23 (29%) of the 78 neurosurgical patients were diagnosed post-surgery with DVT that developed on 2-11th postoperative days. DVT was confirmed by duplex ultrasonography of the lower extremities performed with the GE Logiq700 instrument. Patients were placed in a supine position to visualize the femoro-popliteal area and for the distal area patients were standing or placed in the reverse Trendelenburg position. To examine deep veins, they were compressed by the transducer in the cross-sectional view. Luminal filling defects were revealed using color flow. To see spontaneous flow and phasicity, Doppler tracings were recorded. DVT was diagnosed based on the following criteria: (i) the absence of collapse or only partial collapse of the vein lumen in response to compression; (ii) direct imaging of thrombus inside the vessel; (iii) lack of free blood flow; (iv) lack of Doppler signal; (v) enlarged diameter of the vein. DVT was diagnosed if two or more of these features were revealed. 12 (52%) of the 23 DVT patients had thrombosis of one limb and 11 (48%) patients had thrombosis of both lower extremities. Based on the level of thrombotic occlusion, the patients were segregated to those with distal thrombosis of the femoral-popliteal segment (96% patients) or proximal iliofemoral thrombosis (4% patients). Patients with suspected PE underwent a CT scan of the chest with contrast, but none of the patients examined was diagnosed with PE at the time of examination.

### Blood collection and processing

Blood from each patient was collected and analyzed within 1–3 days prior to surgery and on the 1st day and 5–7th days after the surgery. Venous blood was collected in vacutainers containing 3.2% sodium citrate 9:1 by volume (S-Monovette tubes, Sarstedt, Germany), stored and processed at room temperature and analyzed within 4 h. Citrated blood was divided into two portions: one sample was used for the clot contraction assay and the other sample was centrifuged (1,500 g, 10 min, room temperature) to obtain platelet-poor plasma (PPP) for other blood coagulation tests. Another blood sample was stabilized with K_3_-EDTA (1.6 mg/ml final concentration) and used for hematological tests.

### Blood clot contraction assay

Determination of the kinetics and the extent of clot contraction was carried out using our original method based on the optical detection of clot size over time using the Thrombodynamics Analyzer (HemaCore LLC, Russia)^26^. Citrated blood samples were activated under standard conditions with 1 U/ml human α-thrombin (Sigma-Aldrich, USA) and 2 mM CaCl_2_ (final concentrations). The activated blood samples (80 µl) were quickly transferred to a 12 × 7 × 1 mm transparent plastic cuvette that was pre-coated with a thin layer of 4 v/v% Triton X-100 solution in 150 mM NaCl to prevent sticking of the clot to the walls of the chamber. The transparent cuvette was placed into the temperature-controlled chamber of the Thrombodynamics Analyzer instrument at 37 °C. Images of the contracting clots were taken every 15 s over 20 min to track changes in the clot size based on the light scattering properties of the clot through the use of a light emitting diode and a CCD camera (Fig. 1A). Serial images of the shrinking clot were converted automatically into a kinetic curve of clot contraction (Fig. 1B), from which the following parameters were derived computationally: (1) the extent of contraction (percent), i.e. the degree of shrinkage of the clot after 20 min calculated as [(initial clot size—final clot size)/(initial clot size)]; (2) lag time (seconds), i.e. the time from the addition of thrombin until the clot reaches 95% of its initial size; (3) the area under the kinetic curve (a.u.), which reflects the amount of mechanical work on clot compression performed by the contracting platelets; (4) the average contraction velocity (percent per second) is the extent of clot contraction after 20 min per time. The results obtained at each time point (1–3 days before and on the 1st day and 5–7th days after the surgery) were compared between two subgroups of patients, those with and without retrospectively diagnosed postoperative DVT.

### Coagulation and hematological tests

Hemostasis was evaluated in freshly obtained citrated blood plasma with a Sysmex CA-1500 analyzer (Sysmex, Canada) based on prothrombin time, thrombin time, activated partial thromboplastin time (aPTT), and the level of fibrinogen (Table S6). An immunochemical assay system, IMMULITE 2000 (Siemens Healthcare Diagnostics Inc., United States) was used to measure D-dimer. Hematological analyses were performed in whole blood samples treated with EDTA using an ABX Pentra 60 instrument (Horiba, Japan). The determined hematological parameters are presented in Table S7. The results were compared between two subgroups of patients, those with and without retrospectively diagnosed postoperative DVT.

### Statistical analysis

Statistical analyses were performed using the GraphPad Prism 8 package (GraphPad Software). The normality of data distribution was assessed with the Kolmogorov–Smirnov and Shapiro–Wilk criteria. Statistical differences were estimated using a 95% confidence interval (CI) of the difference between means or medians and a two-way ANOVA followed by Tukey's multiple comparisons post hoc test; the Mann–Whitney U test was also used for pairwise comparisons. The confidence level was 95%. Correlation analysis was performed using the Spearman's rank correlation coefficient. The sensitivity, specificity, positive and negative predictive values, positive and negative likelihood ratios were calculated using a MedCalc statistical package (version 19.2.0).

## Supplementary information

Supplementary Information.
