# Supplementary material for: Impaired contraction of blood clots precedes and predicts postoperative venous thromboembolism
Source: Sci Rep. 2020 Oct 26;10:18261. doi: 10.1038/s41598-020-75234-y (PMC7589563; doi:10.1038/s41598-020-75234-y)
Supplement: Supplementary file 1 — Supplementary Information. [file 41598_2020_75234_MOESM1_ESM.docx]

**SUPPLEMENTARY INFORMATION**

**Impaired Contraction of Blood Clots Precedes and Predicts Postoperative Venous Thromboembolism**

Natalia G. Evtugina^1^, Alina D. Peshkova^1^, Arseniy A. Pichugin^2^, John W. Weisel^3^, Rustem I. Litvinov^1,3^

^1^Institute of Fundamental Medicine and Biology, Kazan Federal University, Kazan,

Russian Federation

^2^Department of Neurosurgery, Interregional Clinical Diagnostic Center, Kazan, Russian Federation

^3^Department of Cell and Developmental Biology, University of Pennsylvania School of Medicine, Philadelphia, Pennsylvania, United States

**Supplemental Figures**


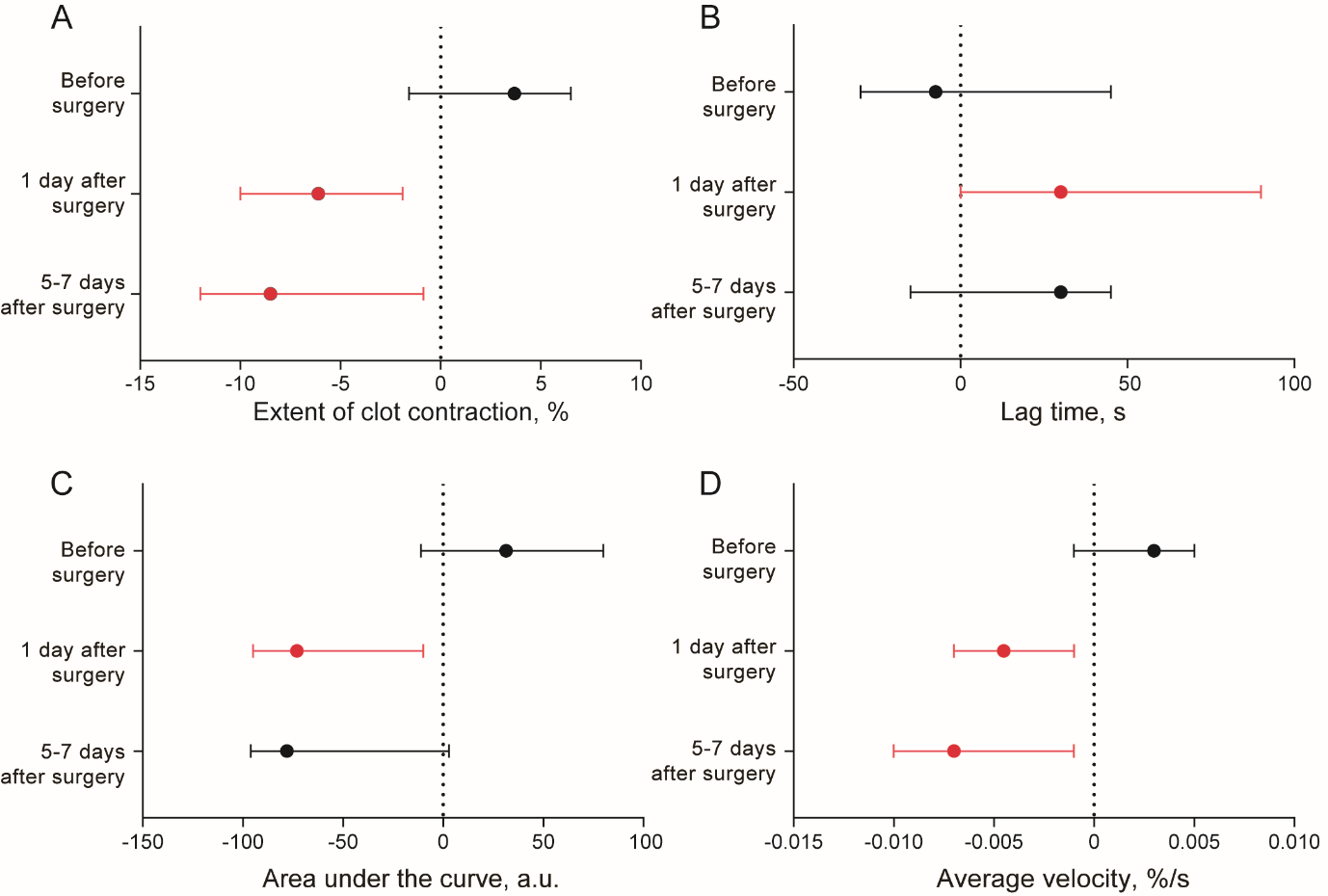


**Fig. S1.** Graphical representation of the differences between medians (dots) and 95% CIs of the differences between medians for parameters of blood clot contraction in neurosurgical patients without and with postoperative DVT before and after surgery. The significant differences are highlighted in red.


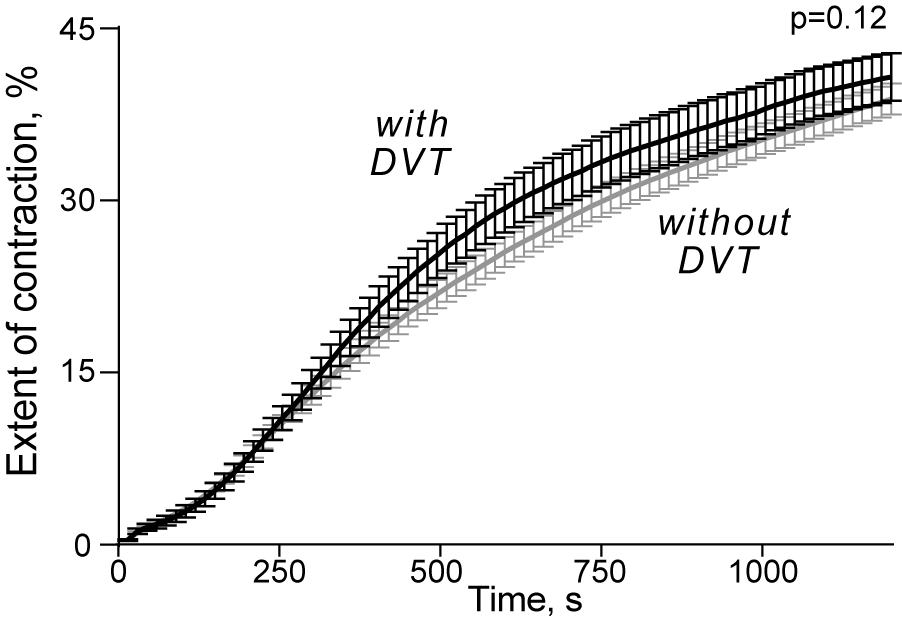


**Fig. S2.** Comparative averaged kinetic curves of clot contraction for patients without and with postoperative DVT examined *1-3 days before the operation*. There were no significant differences between the curves in overall kinetics as well as in durations and rate constants of each phase after fitting the curves with a piecewise function [*Tutwiler et al. Kinetics and mechanics of clot contraction are governed by the molecular and cellular composition of the blood. Blood 2016;127:149-159*].

**Supplemental Tables**

**Table S1.** Parameters of blood clot contraction in neurosurgical patients without (n=55) and with (n=23) postoperative DVT before and after surgery.

| *Parameters* | *Before surgery* | | *1 day after surgery* | | *5-7 days after surgery* | |
| --- | --- | --- | --- | --- | --- | --- |
|  | *Patients without* *DVT* | *Patients with*  *DVT* | *Patients without* *DVT* | *Patients with*  *DVT* | *Patients without* *DVT* | *Patients with*  *DVT* |
| Extent of contraction, % | 40.0  (36.3; 42.3) | 43.7  (36.1; 47.0) | 43.3  (35.2; 45.3) | 37.2*  (30.6; 39.6) | 44.0  (39.8; 45.9) | 35.5*  (28.2; 44.0) |
| Lag time, sec | 165  (135; 180) | 150  (135; 195) | 195  (165; 210) | 225*Ω  (195; 300) | 180  (165; 195) | 210  (150; 240) |
| AUC, a.u. | 274  (254; 301) | 307  (261; 376) | 299  (263; 329) | 226* Ω  (192; 285) | 311  (273; 331) | 232  (203; 320) |
| Average velocity, %/sec | 0.033  (0.029; 0.035) | 0.036  (0.027; 0.038) | 0.036  (0.028; 0.037) | 0.031*  (0.025; 0.033) | 0.036  (0.033; 0.038) | 0.029*  (0.023; 0.037) |

Results are presented as a median with 95% CI in parenthesis.

*p<0.05 between patients without and with postoperative DVT at the same time point,
Ωp<0.01 between patients with postoperative DVT before surgery and 1st day after surgery (2-way ANOVA with Tukey's multiple comparisons post hoc test).

**Table S2.** Differences between medians (and 95% CIs of the difference between the medians) for parameters of blood clot contraction in neurosurgical patients without and with postoperative DVT before and after surgery.

| *Parameters* | *Before surgery* | *1 day after surgery* | *5-7 days after surgery* |
| --- | --- | --- | --- |
| Extent of contraction, % | 3.7  (-1.6; 6.5) | -6.1  (-10;-1.9) | -8.5  (-12.0; -0.9) |
| Lag time, sec | -7.5  (-30; 45) | 30  (0;90) | 30  (-15;45) |
| AUC, a.u. | 31  (-11;80) | -73  (-95;-10) | -78  (-96;3) |
| Average velocity, %/sec | 0.003  (-0.001;0.005) | -0.005  (-0.007;-0.001) | -0.007  (-0.01; -0.001) |

**Table S3.** Extent of clot contraction in patients without and with postoperative DVT in various clinical subgroups *on the 1^st^ day after surgery*

| ***1 day after operation***  *Clinical characteristics* | | *Total number of surgical patients,*  *n=78* | *Clinical subgroups* | |
| --- | --- | --- | --- | --- |
|  |  |  | *Without postoperative DVT,*  *n=55* | *With*  *postoperative DVT,*  *n=23* |
| *Primary diagnosis* | | | | |
| Type of tumor | Malignant | 22 (28%) | 43  (38;47) | 32*  (25;39) |
|  | Benign | 56 (72%) | 38  (35;42) | 35  (31;40) |
| *Comorbidities* | | | | |
| Atherosclerosis | | 61 (78%) | 40  (38;43) | 34**  (31;37) |
| Hypertension | | 32 (41%) | 43  (39;46) | 35**  (31;38) |
| Heart failure | | 21 (27%) | 40  (35;46) | 34  (29;38) |
| Chronic obstructive pulmonary disease | | 6 (8%) | 46  (33;59) | 35  (27;44) |
| Diabetes | | 9 (12%) | 44  (38;50) | 38  (23;54) |
| *Risk factors for thrombosis* | | | | |
| Operative time | < 4 hours | 55 (71%) | 41  (38;43) | 33**  (29;37) |
|  | > 4 hours | 23 (29%) | 38  (36;41) | 33*  (32;41) |
| Obesity (BMI > 30 kg/m^2^) | | 20 (26%) | 43  (38;47) | 38  (34;41) |
| Steroids before and during operation  (dexamethasone, hydrocortisone) | | 36 (46%) | 42  (39;46) | 35*  (31;39) |

Results in the clinical subgroups are presented as a mean with 95% CI in parenthesis.

*p<0.05; **p<0.01 between the patients without and with postoperative DVT (Mann-Whitney U test)

**Table S4**. Extent of clot contraction in patients without and with postoperative DVT in various clinical subgroups on *5-7th postoperative days*.

| ***5-7 days*** ***after operation***  *Clinical characteristics* | | *Total number of operated patients, n=78* | *Clinical subgroups* | |
| --- | --- | --- | --- | --- |
|  |  |  | *Without postoperative DVT, n=55* | *With*  *postoperative DVT, n=23* |
| *Primary diagnosis* | | | | |
| Type of tumor | Malignant | 22 (28%) | 41  (37;46) | 39  (26;53) |
|  | Benign | 56 (72%) | 42  (39;45) | 34*  (28;39) |
| *Comorbidities* | | | | |
| Atherosclerosis | | 61 (78%) | 43  (40;46) | 35**  (29;41) |
| Hypertension | | 32 (41%) | 41  (36;45) | 40  (33;47) |
| Heart failure | | 21 (27%) | 45  (41;49) | 37*  (28;45) |
| Chronic obstructive pulmonary disease | | 6 (8%) | 49  (15;83) | 22  (4;41) |
| Diabetes | | 9 (12%) | 4  (35;49) | 40  (-91;171) |
| *Risk factors for thrombosis* | | | | |
| Operative time | < 4 hours | 55 (71%) | 40  (37;43) | 37  (30;43) |
|  | > 4 hours | 23 (29%) | 45  (40;50) | 34  (16;51) |
| Obesity (BMI > 30 kg/m^2^) | | 20 (26%) | 36  (30;43) | 34  (22;45) |
| Steroids before and during operation (dexamethasone, hydrocortisone) | | 36 (46%) | 44  (40;48) | 34*  (27;43) |

Results in the clinical subgroups are presented as a median with 95% CI in parenthesis.

*p<0.05; **p<0.01 between the patients without and with postoperative DVT (Mann-Whitney U test)

**Table S5.** Characterization of the prognostic power of the clot contraction assay performed on the 1^st^ day after surgery for postoperative DVT at various cut-off levels of the extent of clot contraction.

| *Extent of clot contraction, %* | *Sensitivity,%* | *Specificity,%* | *Positive Predictive Value, %* | *Negative Predictive Value, %* | *Positive Likelihood Ratio* | *Negative Likelihood Ratio* |
| --- | --- | --- | --- | --- | --- | --- |
| <38 | 65  (43;84) | 64  (50;76) | 43  (32;54) | 81  (71;89) | 1.8  (1.1;2.8) | 0.6  (0.3;1.0) |
| <39 | 65  (43;84) | 62  (48;75) | 42  (31;53) | 81  (70;89) | 1.7  (1.1; 2.7) | 0.6  (0.3;1.0) |
| <40 | 87  (66;97) | 60  (46;73) | 48  (39;57) | 92  (79;97) | 2.2  (1.5; 3.1) | 0.2  (0.1;0.6) |
| <41 | 91  (72;99) | 58  (44;71) | 48  (39;56) | 94  (81;98) | 2.2  (1.6;3.1) | 0.2  (0.04;0.6) |
| <42 | 96  (78;100) | 56  (42;70) | 48  (40;56) | 97  (82;100) | 2.2  (1.6;3.0) | 0.1  (0.01;0.5) |
| <43 | 96  (78;100) | 55  (41;68) | 47  (39;54) | 97  (81;100) | 2.1  (1.6;2.9) | 0.1  (0.01;0.6) |
| <44 | 100  (85;100) | 49  (35;63) | 45  (39;52) | 100 | 2.0  (1.5;2.6) | 0 |

Results are presented as a median with 95% CI in parenthesis.

**Table S6.** Hemostatic parameters in neurosurgical patients without (n=55) and with (n=23) postoperative DVT before and after surgery**.**

| *Parameters*  *(in parentheses –*  *reference values)* | *Before surgery* | | *1 day after surgery* | | *5-7 days after surgery* | |
| --- | --- | --- | --- | --- | --- | --- |
|  | *Patients without* *DVT* | *Patients with* *DVT* | *Patients without* *DVT* | *Patients with* *DVT* | *Patients without* *DVT* | *Patients with* *DVT* |
| aPTT  (26-36) sec | 29.5  (27.5; 30.5) | 26.2  (25.3; 29.7) | 28.1  (27.0; 30.0) | 27.4  (24.3; 29.9) | 27.3  (26.1; 28.5) | 27.9  (24.3; 30.0) |
| Fibrinogen  (1.8-4.0) g/L | 3.6  (3.4; 3.9) | 3.5  (2.7; 4.2) | 3.6  (3.2; 3.8) | 3.8  (3.6; 4.1) | 4.1  (3.8; 4.3) | 3.9  (3.6; 4.1) |
| Fibrinogen  (5.3-11.8) μmol /L | 10.6  (10.0; 12.1) | 10.3  (7.9; 12.4) | 10.6  (7.6; 12.4) | 11.2  (10.0; 12.4) | 12.1  (10.3; 12.6) | 11.5  (10.0; 12.6) |
| Thrombin time  (14-21) sec | 18.8  (15.0; 24.5) | 24.2  (19.3; 24.3) | 17.7  (16.6; 19.0) | 16.9  (14.6; 18.7) | 18.6  (14.9; 19.5) | 18.2  (14.3; 22.0) |
| Prothrombin time  (9.8-12.1) sec | 11.4  (11.2; 11.9) | 11.2  (10.6; 11.8) | 12.0  (11.6; 12.1) | 12.0  (11.3; 12.1) | 11.4  (11.2; 11.7) | 11.2  (10.8; 12.3) |
| D-dimer  (0-0.5) μg/mL | 0.2  (0.1; 0.3) | 0.2  (0.1; 1.3) | 1.7  (0.9; 3.7) | 3.0  (0.8; 4.4) | 0.97  (0.8; 1.7) | 2.5*  (0.9; 6.5) |

Results are presented as a median with 95% CI in parenthesis.

*p<0.001 between patients without and with postoperative DVT at the same time point (2-way ANOVA with Tukey's multiple comparisons test)

**Table S7.** Hematologic parameters in neurosurgical patients without (n=55) and with (n=23) postoperative DVT before and after surgery.

| *Parameters*  *(in parentheses –*  *reference values)* | *Before surgery* | | *1 day after surgery* | | *5-7 days after surgery* | |
| --- | --- | --- | --- | --- | --- | --- |
|  | *Patients without* *DVT* | *Patients with* *DVT* | *Patients without* *DVT* | *Patients with* *DVT* | *Patients without* *DVT* | *Patients with* *DVT* |
| Hemoglobin (120-140) g/L | 131  (127; 137) | 130  (101; 137) | 114  (105; 119) | 108  (83; 118) | 116  (110; 125) | 105  (97; 114) |
| Hematocrit  (36-48) % | 38.6  (37.6; 41.4) | 36.4  (28.9; 41.1) | 34.4  (31.7; 36.1) | 32.5  (28.6; 34.6) | 34.8  (32.4; 37.0) | 31.6  (29.4; 34.0) |
| Red blood cells (3.7-4.7) ×10^12^/L | 4.6  (4.3; 4.8) | 4.2  (3.6; 4.7) | 4.1  (3.8; 4.3) | 3.7  (3.3; 3.9) | 4.1  (3.7; 4.2) | 3.6  (3.4; 3.9) |
| Mean corpuscular volume  (80-100) fL | 88  (85; 89) | 89  (86; 92) | 85  (84; 87) | 86  (85; 91) | 87  (86; 88) | 89  (86; 94) |
| Mean cell hemoglobin  (30-35) pg | 29.2  (28.5; 30.2) | 30.3  (28.0; 31.7) | 28.8  (28.4; 39.9) | 29.4  (28.4; 31.4) | 29.4  (28.8; 29.8) | 29.7  (27.8; 30.6) |
| Red cell distribution width  (10-15) % | 13.0  (12.4; 13.2) | 13.1  (11.7; 14.0) | 13.0  (12.7; 13.8) | 13.2  (12.2; 13.9) | 13.1  (12.2; 13.6) | 13.1  (11.5; 14.4) |
| Mean cell hemoglobin concentration (310-360) g/L | 334  (331; 339) | 337  (329; 345) | 338  (333;343) | 338  (332; 351) | 337  (333; 339) | 335  (332; 337) |
| Platelet count (180-320) ×10^9^/L | 235  (208; 248) | 256  (204; 338) | 218  (200; 251) | 229  (183; 300) | 255  (235; 274) | 277  (214; 366) |
| Mean platelet volume  (7.4-10.4) fL | 7.9  (7.5; 8.2) | 7.8  (7.4; 8.5) | 9.1  (7.7; 12.3) | 8.0  (7.6; 12.0) | 8.0  (7.4; 8.6) | 7.8  (7.4; 12.3) |
| Leukocytes count  (4-9) ×10^9^/L | 6.2  (5.6; 6.9) | 6.3  (4.8; 9.6) | 11.0  (9.6; 12.6) | 12.0  (9.0; 15.4) | 7.7  (7.0; 9.8) | 7.1  (6.1; 8.8) |
| Eosinophils  (0.5-5) % | 1.7  (1.1; 3.4) | 1.2  (0.9; 2.3) | 0.8  (0.8; 1.2) | 1.0  (0.8; 1.2) | 2.8  (2.0; 3.4) | 3.1  (1.2; 3.9) |
| Monocytes  (3-11) % | 6.4  (5.4; 6.9) | 6.1  (4.9; 7.0) | 4.9  (4.3; 5.2) | 4.7  (3.8; 6.0) | 6.2  (5.4; 7.1) | 6.2  (5.4; 7.9) |
| Lymphocytes  (19-37) % | 28.8  (23.6; 35.1) | 26.2  (19.4; 34.0) | 11.9  (9.2; 15.6) | 11.6  (8.1; 14.9) | 24.9  (22.2; 27.2) | 24.8  (19.3; 29.5) |
| Basophils  (0-1) % | 0.5  (0.4; 0.7) | 0.4  (0.3; 0.5) | 0.5  (0.4; 0.5) | 0.4  (0.3; 0.5) | 0.6  (0.5; 0.7) | 0.6  (0.3; 0.7) |
| Neutrophils  (47-78) % | 60.2  (52.4; 67.5) | 65.9  (50.8; 78.7) | 76.4  (72.7; 81.6) | 82.2  (75.8; 81.6) | 62.5  (57.7; 69.4) | 61.4  (54.5; 71.1) |

Results are presented as a median with 95% CI in parenthesis.

p>0.05 between the patients without and with postoperative DVT at the same time point (2-way ANOVA with Tukey's multiple comparisons post hoc test)
